# Learning a Model of Shape Selectivity in V4 Cells Reveals Shape Encoding Mechanisms in the Brain

**DOI:** 10.1523/JNEUROSCI.1467-22.2023

**Published:** 2023-05-31

**Authors:** Paria Mehrani, John K. Tsotsos

**Affiliations:** ^1^Department of Electrical Engineering and Computer Science, York University, Toronto, Ontario M3J 1P3, Canada; ^2^Center for Vision Research, York University, Toronto, Ontario M3J 1P3, Canada

**Keywords:** learning shape, shape selectivity, sparse coding, V4

## Abstract

The mechanisms involved in transforming early visual signals to curvature representations in V4 are unknown. We propose a hierarchical model that reveals V1/V2 encodings that are essential components for this transformation to the reported curvature representations in V4. Then, by relaxing the often-imposed prior of a single Gaussian, V4 shape selectivity is learned in the last layer of the hierarchy from Macaque V4 responses. We found that V4 cells integrate multiple shape parts from the full spatial extent of their receptive fields with similar excitatory and inhibitory contributions. Our results uncover new details in existing data about shape selectivity in V4 neurons that with additional experiments can enhance our understanding of processing in this area. Accordingly, we propose designs for a stimulus set that allow removing shape parts without disturbing the curvature signal to isolate part contributions to V4 responses.

**SIGNIFICANCE STATEMENT** Selectivity to convex and concave shape parts in V4 neurons has been repeatedly reported. Nonetheless, the mechanisms that yield such selectivities in the ventral stream remain unknown. We propose a hierarchical computational model that incorporates findings of the various visual areas involved in shape processing and suggest mechanisms that transform the shape signal from low-level features to convex/concave part representations. Learning shape selectivity from Macaque V4 responses in the final processing stage in our model, we found that V4 neurons integrate shape parts from the full spatial extent of their receptive field with both facilitatory and inhibitory contributions. These results reveal hidden information in existing V4 data that with additional experiments can enhance our understanding of processing in V4.

## Introduction

Transformation of the shape signal in the ventral stream from low-level visual representations of V1 (oriented edges) (Hubel and Wiesel, 1959; Hubel, 1963) and V2 (corners and junctions) (Hegdé and Van Essen, 2000; Ito and Komatsu, 2004) to more abstract representations in IT (objects, faces, etc.) (Tanaka, 1996; Kobatake et al., 1998) is still unknown. V4, as an intermediate processing stage in this pathway and as the major source of input to IT, is believed to play a role in this transformation (Roe et al., 2012; Pasupathy et al., 2020). Yet, selectivities to shape features in V4 neurons add to this mystery. Specifically, with many V4 neurons selective to convex and concave shape parts at a specific position relative to the object center (Pasupathy and Connor (1999, 2001, 2002), it remains unclear how such part-based and object-centered curvature representations are achieved in the ventral stream. Here, we tackle this problem in two steps: (1) transformation of the shape signal into an object-center curvature encoding in the ventral stream; and (2) part-based selectivity in V4.

### Step 1: signed curvature encoding

**Figure 1.**
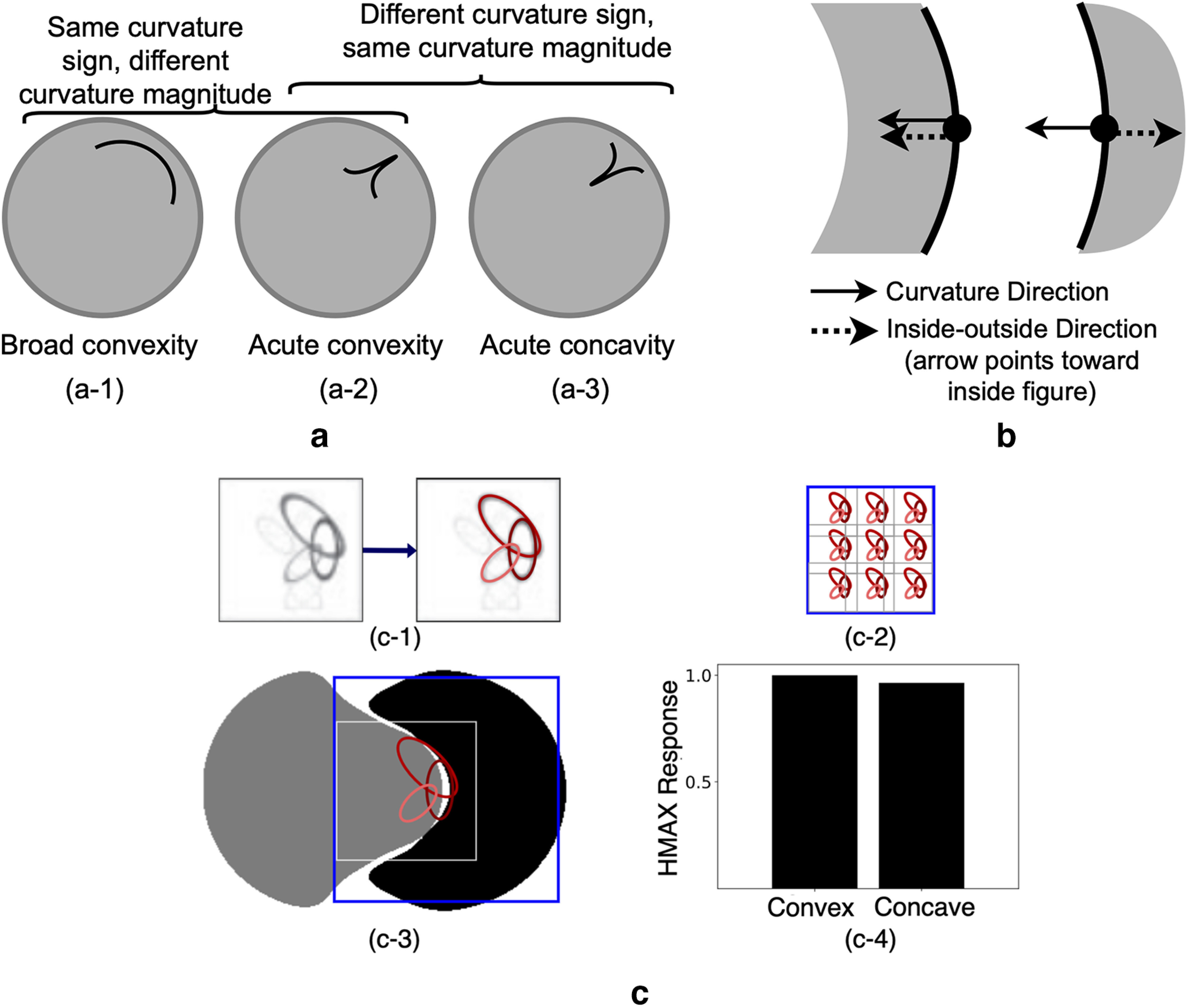
Signed curvature selectivity in V4. ***a***, Signed curvature encapsulates two curvature components: magnitude and sign. Here, curved segments in ***a-1*** and ***a-3*** share either curvature magnitude or sign with the one in ***a-2***. A V4 cell selective to acute convexities on the top right side of its RF will be strongly activated by the stimuli in ***a-2*** and not ***a-1*** or ***a-3***. ***b***, Conventionally, inside-outside direction for a closed simple planar shape defines a unique signed curvature for each point on its bounding contour. Even with identical curvature magnitude and direction, the same curved segment might be a convex or a concave part of a closed shape. One such example is demonstrated here for the two gray shapes with identical curve segments that are denoted with solid black curves. For a simple closed shape, with matching curvature direction and inside-outside direction, the curve is considered convex. Otherwise, it is concave. ***c***, Lack of curvature sign representations in previous V4 models. ***c-1***, The S2 configuration of a HMAX C2 unit is shown in the left square (extracted from Cadieu et al., 2007). In HMAX, responses of C2 units simulating V4 responses are obtained by a max operation over a 3 × 3 map of overlapping S2 units. Here, each oval represents an orientation-selective C1 afferent (for details, see Cadieu et al., 2007) with its perimeter intensity indicating its weight to the S2 cell. For simplicity, we retained three components with the largest weights as shown in the right square. ***c-2***, The 3 × 3 C2 grid with overlapping S2 afferents. ***c-3***, We simulated an HMAX C2 cell with the S2 afferent shown in ***c-1*** and recorded its responses to two shapes with convex/concave parts placed at the center of its RF. Blue square represents the simulated V4 RF and its S2 unit with the strongest contribution to its responses to these shapes. The filling colors of the shapes are only for demonstration. ***c-4***, Normalized responses of the simulated neuron plotted as bars demonstrate comparable responses to convex/concave shape parts at the RF center (i.e., a lack of signed curvature encoding).

**Figure 2.**
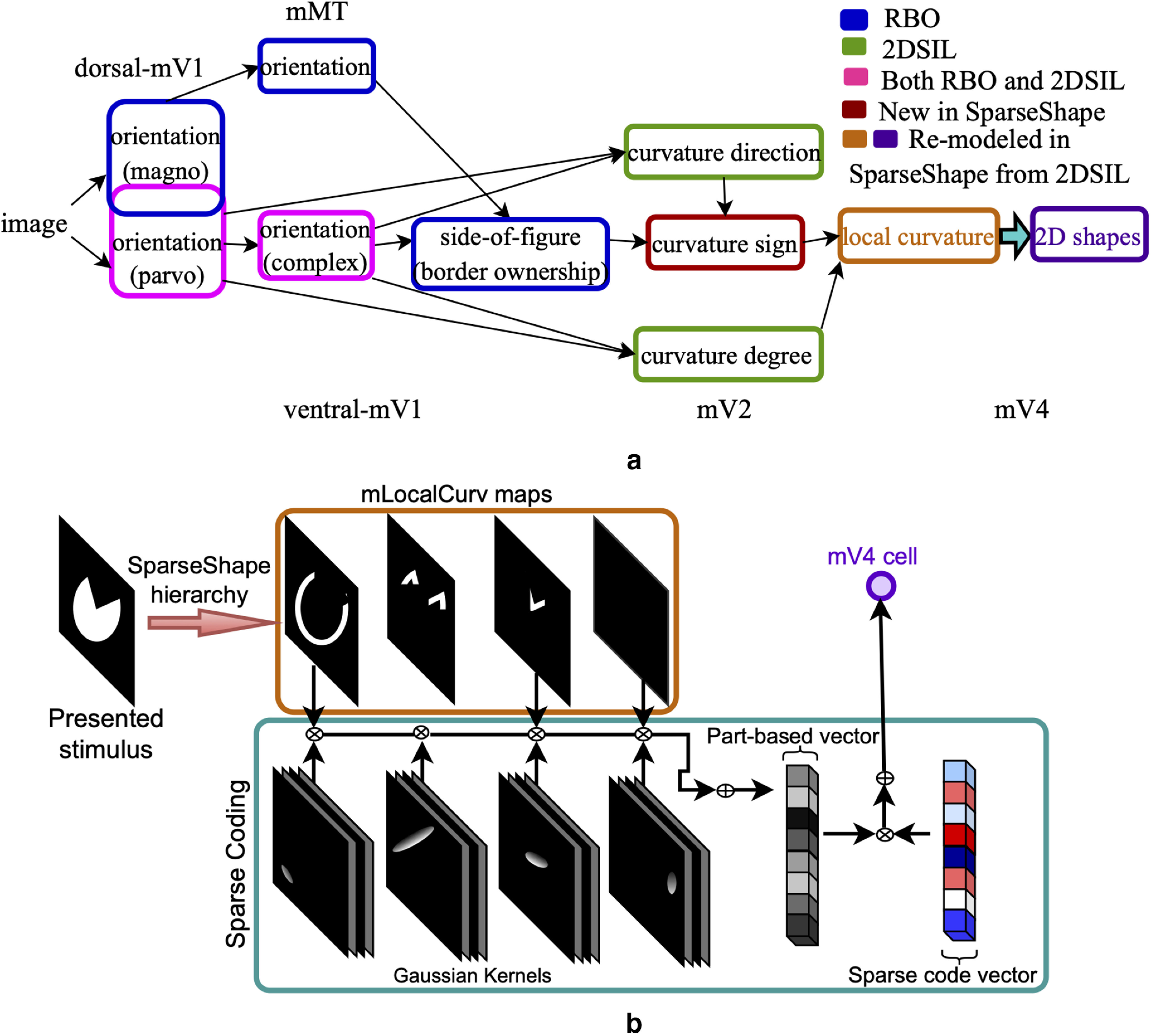
The proposed model. ***a***, The SparseShape hierarchy combines and extends two previously introduced hierarchical models: RBO (Mehrani and Tsotsos, 2021) and 2DSIL (Rodríguez-Sánchez and Tsotsos, 2012). The color of each box represents the origin of the neuron from 2DSIL, RBO, or SparseShape. 2DSIL models curvature magnitude and direction through endstopped neurons. To achieve a signed curvature encoding, inside-outside information is represented by incorporating the RBO network modeling BO. In SparseShape, new neurons modeling curvature sign are added to the network that enable signed curvature selectivity in the mV4 layer. Accordingly, the model local curvature cells are remodeled with feedforward signals from curvature degree and sign cells. Similarly, mV4 cells are remodeled with the sparse coding step. ***b***, The various components of the supervised sparse coding algorithm are shown in the cyan box in this figure corresponding to the cyan arrow in ***a***. Given mLocalCurv maps and Macaque V4 responses to stimuli in the training set, the algorithm looks for a combination of shape conformations that could best explain mV4 responses. To account for the various positions of shape parts, a set of 9 Gaussian kernels spanning a 3 × 3 grid over the RF filter mLocalCurv responses at each cell. The filtered responses form the part-based vector. The sparse code vector denotes the weight of each part contributing to the responses. The parameters of the Gaussian kernels are fixed while the sparse code vector is learned in this step.

Findings of shape processing in V4 provide evidence for selectivity to two curvature components: curvature magnitude (deviation from a straight line) and curvature sign (convexity-concavity, determined according to a set origin), together defining the scalar called signed curvature. Figure 1*a* depicts how two curve segments can share the same curvature sign with different curvature magnitudes or have the same curvature magnitude but different curvature signs. A V4 neuron selective to acute convexities at the top right side of its receptive field (RF) exhibits strong responses to stimuli in Figure 1*a-2* and not to those on either side (for such a tuning in a Macaque V4 cell, see Pasupathy and Connor, 2001, their Fig. 5). The angular position and curvature (APC) model introduced in Pasupathy and Connor (2001) revealed signed curvature selectivity in V4 neurons. Despite the observed selectivities to both curvature magnitude and sign, existing models of V4 encode curvature magnitude but none models curvature sign (for details, see Table 1). Lack of a curvature sign encoding in these models results in responses that are in contrast to the reported observations in V4. Figure 1*c* gives a simple example to demonstrate this disparity. Therefore, when the goal is to understand the development of a signed curvature representation in the ventral stream, these models leave a gap in our understanding of shape signal transformation from orientation encodings to selectivity to a signed curvature representation. This gap is especially evident in the APC model with direct mapping of stimulus shape to the position and signed curvature domain.

**Table 1.** Comparison of previous models for V4 shape representation*^a^*

Model	Long-range interactions	Excitatory/ inhibitory	Modeled ventral areas	Signed curvature encoding	Stimuli set	Model/code available	Comparison with data from Pasupathy and Connor (2001)
**APC–2D, APC–4D** Pasupathy and Connor (2001)	**No**	**No**	**V4**	**Yes**	**Standard set**	**No**	**Yes**
HMAX Cadieu et al. (2007)	Yes	No	V1, V4	No	Standard set	No	Yes
2DSIL Rodríguez-Sánchez and Tsotsos (2012)	Yes	No	V1, V2, V4	No	Standard set	Yes	Yes
Popovkina et al. (2019)	Yes	Yes	V1, V4	No	Synthetic outline/filled shapes	No	No
Hu et al. (2014)	Yes	No	V1, V4	No	Synthetic and natural shapes	No	No
Wang and Deng (2016)	Yes	No	V1, V4	No	Natural images	No	No
Eguchi et al. (2015)	Yes	Yes	V1, V2, V4 (and higher areas)	No	Synthetic and natural shapes	No	No
Wei and Dong (2015)	Yes	Yes	V1, V4	No	Synthetic and natural shapes	No	No
Rodríguez-Sánchez et al. (2016)	Yes	Yes	V1, V2, V4	No	Standard set	No	No
Hatori et al. (2016)	Yes	Yes	V1, V2, V4	No	Natural shapes	No	No
Kawakami et al. (2020)	Yes	No	V1, V4	No	Synthetic shapes	No	No
**SparseShape**	**Yes**	**Yes**	**V1, V2, V4**	**Yes**	**Standard set**	**Yes**	**Yes**

*^a^*Among the models in this table, APC (2D and 4D) was utilized in recovering V4 tunings. Therefore, despite revealing V4 shape representations, it is an input-output mapping and does not provide an explanation on how these representations are achieved in the ventral stream. For this reason, APC is separated from other models with bold font. The rest of the models vary in this regard. For example, HMAX skips modeling neurons in V2 and, hence, encapsulates V2-V4 representations into a single shape-selective model layer, its S2 layer. In contrast, 2DSIL models neurons in V1, V2, and V4. All models, except for APC, account for long-range interactions; and some recover both excitatory/inhibitory contributions. However, none of the previously introduced models provides a formulation for signed curvature encodings in the ventral stream. Among these models, APC, HMAX, and 2DSIL utilized the same stimulus set introduced by Pasupathy and Connor (2001). We call this set as the standard set (see Materials and Methods), which we also used in the present work. Additionally, only the APC, HMAX, and 2DSIL models directly compared their predicted responses with the V4 recordings of Pasupathy and Connor (2001).

### Step 2: part-based selectivity

Pasupathy and Connor (2001) fit boundary conformation tunings in V4 cells with a Gaussian function in the APC space. However, with the strong prior of fitting a single Gaussian to V4 responses, complex and long-range interactions between shape parts within the RF cannot be captured. Fitting two Gaussians with positive and negative weights, Pasupathy and Connor (2001) reported improvement in predicted responses and suggested a “more complex analysis would provide a much better description of shape tunings.” Nevertheless, the single-Gaussian approach has been used in recent V4 studies (El-Shamayleh and Pasupathy, 2016; Popovkina et al., 2019), providing a limited picture of part-based selectivity in V4. With complex response patterns in IT (Brincat and Connor, 2004), we asked whether similar patterns can be observed in V4 cells as a source of input to IT.

In this work, we introduced a hierarchical network modeling neurons in V1, V2 and V4, dubbed SparseShape, that explicitly models both curvature magnitude and sign. Hence, a signed curvature encoding is achieved that could explain the shape signal transformation in the ventral stream. Then, given model signed curvature encodings, we learned shape part combination patterns with no hard constraints on the number of parts, position, or contribution, and investigated the potential impact of including inhibitory shape parts in V4 responses. Specifically, given local curvature cell responses, for each Macaque V4 neuron from the study by Pasupathy and Connor (2001), we used a supervised sparse model to learn a set of contour segments that determine the neuron responses. To the best of our knowledge, SparseShape is the first hierarchical model that provides an explicit signed curvature formulation in the primate ventral visual pathway.

## Materials and Methods

In what follows, we will make a distinction between our model and brain areas by referring to those as layers and areas, respectively. That is, a set of model neurons implementing cells in a brain area will be referred to as a model layer. For example, model layer V2 implements cells in brain area V2. Moreover, whenever a brain area name is preceded with “m,” it is referring to the corresponding model layer, for example, “mV2” for model layer V2.

**Figure 3.**
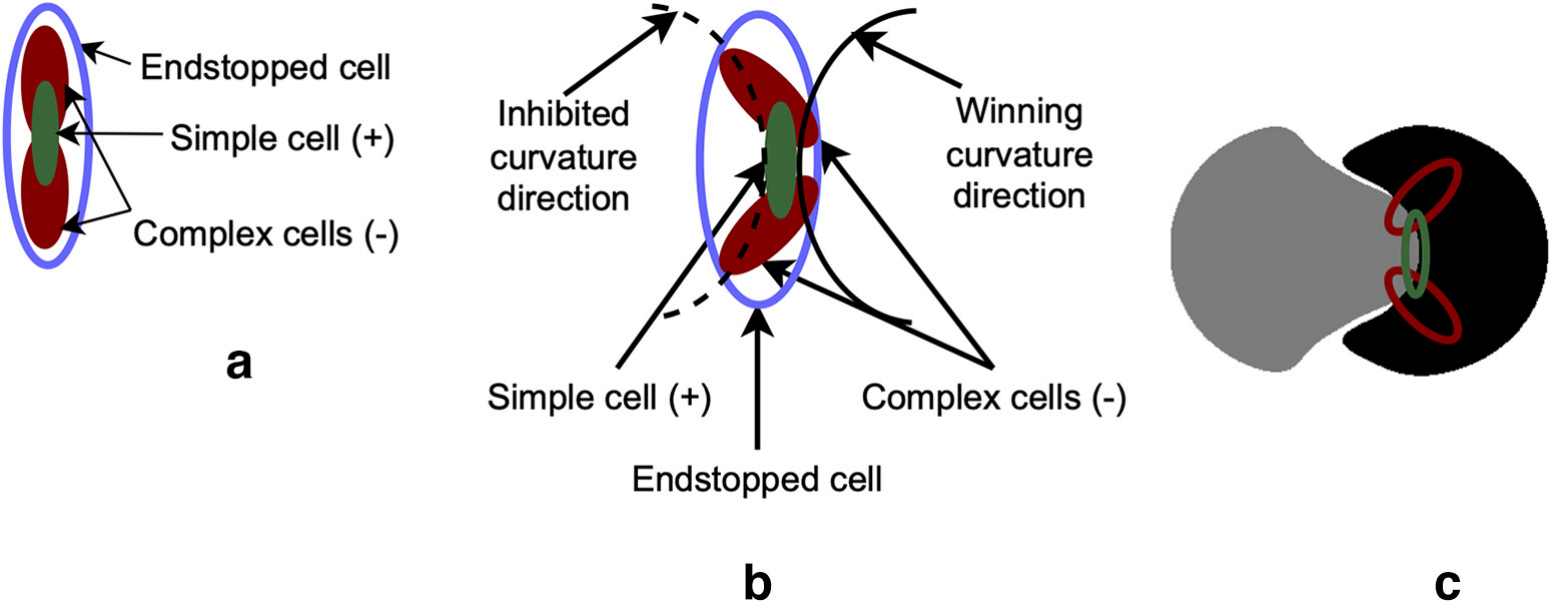
2DSIL endstopping neuron configurations. ***a***, 2DSIL curvature degree neurons receive facilitatory and inhibitory signals from a simple cell and two displaced complex cells, respectively. Positive and negative signs indicate facilitatory/inhibitory contributions. Responses of these neurons encode deviation from a straight line. Combining responses of these cells at four scales represent curvature magnitude. ***b***, The configuration of 2DSIL curvature direction neurons shows a simple cell with two displaced complex cells at orientations 45° and 135° with respect to that of the simple cell. This neuron will exhibit strong responses to the contour segment shown with a solid line, and its responses are inhibited because of inhibition from the pair of complex cells to the dashed contour segment. ***c***, A curvature direction neuron from 2DSIL is drawn on the example from Figure 1*c* to demonstrate the lack of curvature sign encoding in these neurons, similar to the example HMAX cell in Figure 1*c*. Although Rodríguez-Sánchez and Tsotsos (2012) specified these cells as selective to the sign of curvature, this example clearly shows that these neurons only encode curvature direction and will exhibit similar responses to both convex and concave shape parts in these two shapes. Interestingly, the configuration of orientation-selective neurons in 2DSIL endstopping cells looks strikingly similar to that of the HMAX example cell shown in Figure 1*c*, suggesting that S2 cells in HMAX perform endstopping.

SparseShape, whose architecture is depicted in Figure 2*a*, combines and extends two previous models: RBO (Mehrani and Tsotsos, 2021) and 2DSIL (Rodríguez-Sánchez and Tsotsos, 2012). In Figure 2*a*, each model neuron type in the hierarchy is represented with a single box. The color of each box indicates whether the model neuron is borrowed from the RBO, 2DSIL, remodeled from 2DSIL, or new in SparseShape. In SparseShape, simple and complex cells extract oriented edges. These model neurons represented by magenta boxes in Figure 2*a* were implemented in both 2DSIL and RBO with the same formulation and parameter settings that we also used in SparseShape. Complex cell responses modulated by early recurrence from the dorsal stream result in border ownership (BO). Model dorsal neurons and BO cells shown in blue boxes in Figure 2*a* are borrowed from the RBO network. Combining simple and complex cell responses result in two types of endstopped neurons: curvature degree (mEsDeg) and curvature direction (mEsDir). These two types of curvature cells shown in green boxes in Figure 2*a* are adopted following the endstopping implementation in 2DSIL. Figure 3*a* depicts the configuration of simple and complex cells that result in curvature degree endstopped cells that at four scales represent curvature magnitude. Figure 3*b* depicts simple and complex neuron configurations that yield curvature direction representations. Although Rodríguez-Sánchez and Tsotsos (2012) indicated selectivity to curvature sign for neurons in Figure 3*b*, the example in Figure 3*c* with one such neuron superimposed on two shapes with convex-concave parts shows that these cells only signal curvature direction. Hence, we will call them curvature direction cells in what follows.

In SparseShape, we modeled the curvature sign representation according to the observation that it can be uniquely determined for any point on the bounding contour of a simple planar closed shape by combining two signals: inside-outside and curvature direction (i.e., the direction toward which the contour curves; Fig. 1*b*). Neural correlates for both signals in the ventral stream, namely, BO (Zhou et al., 2000) and endstopping (Dobbins et al., 1987; Ito and Komatsu, 2004), support the plausibility of the proposed model. Therefore, curvature sign (mEsSign) encoding is achieved by combining mBO responses with that of curvature direction (mEsDir) neurons. In addition to support from available biological findings, the mBO and mEsDir neurons that give rise to a curvature sign encoding have geometric interpretations that are explained in detail below. Curvature sign (mEsSign) is new in SparseShape compared with 2DSIL, as indicated by the red box in Figure 2*a*. Combining curvature sign and curvature degree signals yield the signed curvature representation manifested in model local curvature (mLocalCurv) neurons, enclosed in an orange box in Figure 2*a*, that are remodeled from the original 2DSIL network. In 2DSIL, model local curvature cells combined curvature direction and curvature degree responses and therefore did not encode signed curvature. In SparseShape, however, a proper signed curvature encoding is achieved in these remodeled cells. Finally, model local curvature cells feed their signal to model shape-selective cells in the output layer of SparseShape representing V4 selectivities. The set of weights in this final stage is learned by using a supervised sparse coding algorithm, replacing the heuristic approach used in 2DSIL.

The SparseShape network meets known biological properties with its parameters set according to neurophysiological findings. Our model implements edge- and border-selective neurons at 12 orientations in (0, π) and combines responses of mBO neurons with edge and border selectivity at the same orientation to a single inside-outside signal that is fed to mEsSign cells. Following 2DSIL and RBO, SparseShape implements neurons at 4 scales that result in 8 mLocalCurv maps (4 scales representing curvature magnitude × 2 signs).

Additionally, neurons up to and including mBO, mEsDeg, and mEsDir are the same as those in RBO and 2DSIL, the computational details of which can be found elsewhere (Rodríguez-Sánchez and Tsotsos, 2012; Mehrani and Tsotsos, 2021). Below are outlines of the computations of new and remodeled neurons in SparseShape and the supervised learning step.

### Model endstopped: curvature sign

Intuitively, a contour segment of a simple closed curve is convex when the contour segment curves toward inside the shape. Figure 1*b* gives examples for which curvature direction and inside-outside information determine curvature sign for contour segments as parts of two shapes. Both curvature direction and inside-outside information have geometric interpretations (for details, see Pressley, 2010): for each point on a contour segment, curvature direction denotes the direction of the tangent vector derivative, whereas inside-outside information represents the direction of the unit normal to the curve. When the tangent derivative and unit normal have similar directions, the signed curvature is positive and the curve is convex; otherwise, the contour segment is concave (assuming exclusion of inflection points). In our proposed hierarchy, curvature direction and inside-outside signal are modeled in mEsDir and mBO cells, together making the curvature sign modeling possible in this network.

In SparseShape, at each visual field location, a pair of mBO neurons with identical local feature selectivity but opposite side-of-figure preferences are modeled. Similarly, a pair of mEsDir with opposite direction selectivities but identical orientation are modeled. Between each pair, the neuron with stronger response is called the winning cell, for example, the winning mBO neuron. When the direction of the winning mEsDir and mBO neurons is in agreement, the contour segment is convex and concave otherwise, resulting in the following implementation of mEsSign cells:

(1) $$R_{\text{mEsSign}\_\text{convex}}(x,y,\theta )=\phi ((R_{\text{mEsDir}}(x,y,\theta +\frac{\pi }{2})-R_{\text{mEsDir}}(x,y,\theta -\frac{\pi }{2}))\times (R_{\text{mBO}}(x,y,\theta +\frac{\pi }{2})-R_{\text{mBO}}(x,y,\theta -\frac{\pi }{2}))),R_{\text{mEsSign}\_\text{concave}}(x,y,\theta )=\phi (-(R_{\text{mEsDir}}(x,y,\theta +\frac{\pi }{2})-R_{\text{mEsDir}}(x,y,\theta -\frac{\pi }{2}))\times (R_{\text{mBO}}(x,y,\theta +\frac{\pi }{2})-R_{\text{mBO}}(x,y,\theta -\frac{\pi }{2}))),$$

where $\left(R_{\text{mEsDir}}(x,y,\theta +\frac{\pi }{2})-R_{\text{mEsDir}}(x,y,\theta -\frac{\pi }{2})\right)$ and $\left(R_{\text{mBO}}(x,y,\theta +\frac{\pi }{2})-R_{\text{mBO}}(x,y,\theta -\frac{\pi }{2})\right)$ denote the direction of winning mEsDir and mBO neurons and the multiplication tests their direction alignment. *R_x_* represents the response of neuron type *x* with x∈{mEsSign_convex,mEsSign_concave,mEsDir,mBO} and θ denotes the orientation selectivity of the corresponding mEsDir or mBO cell. The function ϕ rectifies negative responses to zero.

### Model local curvature

Similar to mEsDir and mEsSign cells, signed curvature is encoded by a pair of mLocalCurv neurons at each visual field location to represent positive and negative signs for a given curvature magnitude. Specifically, at each scale in the hierarchy, a single map of mEsDeg and a pair of mEsSign (mEsSign_convex and mEsSign_concave) are combined to yield pairs of mLocalCurv cells as follows:

(2) $$R_{\text{mLocalCurv}\_\text{pos}}(x,y)=\overset{12}{\underset{j=1}{\max}}(R_{\text{mEsDeg}}(x,y,\theta_{j})\cap (R_{\text{mEsSign}\_\text{convex}}(x,y,\theta_{j})>R_{\text{mEsSign}\_\text{concave}}(x,y,\theta_{j}))),R_{\text{mLocalCurv}\_\text{neg}}(x,y)=\overset{12}{\underset{j=1}{\max}}(R_{\text{mEsDeg}}(x,y,\theta_{j})\cap (R_{\text{mEsSign}\_\text{convex}}(x,y,\theta_{j})<R_{\text{mEsSign}\_\text{concave}}(x,y,\theta_{j}))).$$

where “max” retains responses to curvature at this level of the hierarchy and marginalizes orientation. Consequently, the final layer feeding to mV4 neurons consists of 8 curvature maps, 4 scales × 2 signs, representing four levels of acuteness for convexities and concavities.

### Model V4: learning RFs

Our goal was to learn V4 RFs such that complex and long-range interactions between shape parts, if they exist, can be captured. Recovering existence or equivalently lack of such interactions in V4 imparts significant insight into shape processing mechanisms in this visual area.

We trained the weights in the last layer of SparseShape, mLocalCurv to mV4, and recovered the RFs from the recordings provided to us by Dr. Anitha Pasupathy. Briefly, for each Macaque V4 cell, we assigned its responses to an mV4 cell in SparseShape and learned the weights from mLocalCurv cells to each mV4 neuron. That is, the procedure for learning the RF was repeated for each individual mV4 cell.

A naively-added, fully-connected layer from mLocalCurv to mV4 cells has more than 14K weights to learn from <366 data points. To compensate for the imbalance between the number of parameters and data, we propose imposing sparsity priors that are compatible with discoveries of V4 (Carlson et al., 2011) and other brain areas involved in shape representation (Tsunoda et al., 2001). We leverage sparsity in a higher dimensional space and with a more relaxed model compared with APC. With the learned RFs, obtaining mV4 responses to any arbitrary stimulus set, such as shapes in the invariance experiment explained in Experimental design, is a simple feedforward pass (with dorsal recurrence) in SparseShape.

Our proposed sparse coding method formulates RF recovery as a supervised learning problem. Specifically, given the responses of mLocalCurv cells and Macaque V4 responses to a stimulus set, we seek a sparse combination of curvature components across the RF that can explain observed responses by minimizing the following objective function:

(3) $$\underset{\gamma }{\min}\sum_{t=1}^{\tau }\frac{1}{2}||R_{t}-D_{t}^{T}\gamma |{|}_{2}+\alpha \rho ||\gamma |{|}_{1}+\frac{\alpha (1-\rho )}{2}||\gamma |{|}_{2}^{2},$$

where *R_t_* is the Macaque V4 response to stimulus *t* and τ is the number of stimuli in the training set. This objective function, known as the elastic net model, combines L1 and L2 penalties to ensure a sparse representation with regularized learned weights. Also, the sparsity constraint enforces stability in weights across the various iterations of optimization. In this equation, *D_t_* is the part-based vector whose elements signal presence/absence of a shape part at a particular position within the RF and γ is the sparse code vector that specifies the weight of each curvature component contributing to responses. The sign of each element in γ determines facilitatory versus inhibitory contribution. The trade-off between L1/L2 norms and the error term is enforced through α. ρ determines the balance between L1 and L2 norms. Both α and ρ are set with cross-validation.

The part-based vector *D_t_* is obtained by a set of Gaussian kernels that filter mLocalCurv maps. In particular, to account for a variety of shape part positions, a 3 × 3 grid over the RF filters each mLocalCurv map. Each cell of this grid encompasses a Gaussian kernel. The parameters of each Gaussian characterize the position and extent of a particular curvature component within a cell. Putting these together, $d_{t}(i)$, the *i*-th element of *D_t_*, is computed as follows:

(4) $$d_{t}(i)=\sum_{x,y}C(x,y,t,k)\cdot G(x,y,j;\mu_{j},\Sigma_{j}),$$

with C(x,y,t,k) corresponding to the responses in the *k*-th mLocalCurv map to stimulus *t* at visual location (*x*, *y*) with k∈{1,2,...,8}. In this equation, $G(x,y,j;\mu_{j},\Sigma_{j})$ represents the Gaussian in the *j*-th grid cell with parameters $\mu_{j},\Sigma_{j}$ and j∈{1,2,...,9}. We explored learning the parameters of the Gaussian kernels for each neuron. However, we did not find any benefits in learning these kernels compared with fixing those to predetermined kernels. As such, we fixed the parameters of the Gaussians in this work. With 8 mLocalCurv maps and 9 cell positions, *D_t_* and γ in our implementation are 72-dimensional vectors.

Having found the desired curvature components and their weights, responses of the mV4 neurons corresponding to a given Macaque V4 cell to an arbitrary stimulus *s* can be obtained by:

(5) $$R_{\text{mV4}}=D_{s}^{T}\gamma .$$

The diagram in Figure 2*b* shows the different components of our sparse coding formulation. The cyan box in this figure corresponds to the cyan arrow in Figure 2*a*.

### Experimental design

We conducted experiments with two sets of stimuli: 366 parametric shapes combining convex-concave parts into closed shapes borrowed from Pasupathy and Connor (2001) and parametric shapes with a single varying part along its contour designed according to the set from El-Shamayleh and Pasupathy (2016). The latter set was used to test invariance in mV4 responses. The former shape set, which we call the standard set, was used in all other experiments as well as training the model. The standard set shapes were scaled such that stimulus edges were offset from the RF center by 0.75 × RF size. The shapes in the invariance experiments were scaled following El-Shamayleh and Pasupathy (2016). The SparseShape network input was set to 400 × 400 pixels, and we measured 1° visual angle at 50 cm to be 32 × 32 pixels. We set mV4 RFs at 4° following Felleman and Van Essen (1991) equivalent to 128 × 128 pixels.

Supervised learning in the last layer of SparseShape was performed with electrophysiological responses of 109 Macaque V4 neurons reported by Pasupathy and Connor (2001). That is, for each Macaque V4 cell, we assigned its responses to an mV4 neuron and learned the model weights from mLocalCurv cells to the mV4 neuron. A stratified division of the standard shape set into 60%-40% train and test splits was performed for each individual neuron to ensure an assortment of responses were present in each set. A stratified split has the advantage of reducing sensitivity to the amount of training data. The hyperparameters for learning the last layer weights were determined using cross-validation over the training set followed by learning the weights with all the training shapes. All other model parameters were set according to experimental findings (Rodríguez-Sánchez and Tsotsos, 2012; Mehrani and Tsotsos, 2021).

### Visualizing learned RFs

Visualizing the learned RFs provides a powerful pictorial tool in revealing shape tuning. Here, we provide a brief explanation of how learned RFs can be visualized as depicted in Figure 7*b*. Given the mV4 response formulation in Equations 4 and 5, shape tuning in mV4 cells is determined according to three main components: model local curvature neurons signaling presence/absence of contour segments of a particular signed curvature, the Gaussian kernels specifying position/extent of contour segments within the RF, and the learned sparse code vector. In SparseShape, mLocalCurv neurons are analytically defined; therefore, their selectivity is independent of mV4 responses. Accordingly, for each mLocalCurv map in the model, we first determined the contour segment in the standard stimuli dataset that invokes the strongest responses for neurons in the map. Then, for each mLocalCurv map, we create an auxiliary map by replicating this isolated contour segment in each cell of a 3 × 3 grid identical to the one we used in computing the part-based vector (Eq. 4). Each auxiliary map is then convolved by the set of Gaussian kernels as specified in Equation 4 and multiplied by its corresponding learned weights in the sparse code vector γ. Finally, a summation of all of these maps yields the learned RF.

### Model comparison

Our goal was to understand signed curvature encodings and part-based selectivities in V4 neurons. This goal can be best achieved by training and evaluating the model with available neural data. Therefore, we conducted this study with responses of Macaque V4 neurons reported in Pasupathy and Connor (2001), provided to us by Pasupathy. Specifically, we trained and evaluated our model according to responses of Macaque V4 neurons. To measure model performance, we evaluate individual mV4 cell predictions following Pasupathy and Connor (2001). That is, we report Pearson's *r* correlation coefficients between predicted and observed Macaque V4 responses, separately for train and test sets. Although correcting for trial-to-trial noise could afford an unbiased estimate in reported correlations (Pospisil and Bair, 2021), the Macaque V4 responses we used in our study consisted of a single response per neuron per shape in the standard dataset with no noise analysis incorporated. In other words, with the available data, there was no flexibility to evaluate performance on a trial-to-trial basis and with noise. Therefore, following Pasupathy and Connor (2001) and Cadieu et al. (2007), we report correlation coefficients. Additionally, we report mean absolute error (MAE) of responses following Rodríguez-Sánchez and Tsotsos (2012).

We compare our model performance against two previous hierarchical models for V4, namely, 2DSIL (Rodríguez-Sánchez and Tsotsos, 2012) and HMAX (Cadieu et al., 2007). Among previous hierarchical models (Table 1), these two models are closely related to our proposed model with direct comparison with Macaque V4 data from the study by Pasupathy and Connor (2001) that we also used for training/testing our mV4 cells. Additionally, both models used the same stimulus set as SparseShape, the standard set, for their experiments and evaluation. Both 2DSIL and HMAX modeled curvature magnitude and direction and not curvature sign. Both models used heuristic approaches to determine weights representative of V4 selectivities in their hierarchies. HMAX used a greedy algorithm to recover V4 RFs (see Cadieu et al., 2007, their Fig. 9), while shape templates in 2DSIL were computed based on common shape parts among each neuron's preferred stimuli. In HMAX, the recovered RFs do not necessarily result in curvature-like configurations; whereas in 2DSIL, having a shape template is not guaranteed (they reported results for 75 of 109 Macaque V4 cells).

Alongside HMAX and 2DSIL, we compare our model performance with that of the APC model (Pasupathy and Connor, 2001). APC represents signed curvature and thus is most similar to SparseShape, but it was proposed to recover tunings rather than to explain mechanisms. As such, it is important to keep in mind that, whereas our goal was to understand the transformation of the signal in a step-by-step manner to a signed curvature encoding, the APC model and its variants (Popovkina et al., 2019) bypass crucial steps in this transformation and perform a direct mapping of stimulus shape to the position and signed curvature domain to recover tunings.

In addition to fitting the tunings in the two-dimensional APC space (we term this the APC–2D model), Pasupathy and Connor (2001) evaluated a four-dimensional APC model (three curvatures and one angular-position) by considering the two neighboring curvature components on either side of a shape part. We call this model APC–4D. It is worth mentioning that Pasupathy and Connor (2001) also modeled V4 responses with two Gaussian peaks in the 2D space of curvature and angular position to recover complex tuning functions. In this model, they allowed both positive and negative amplitudes for the Gaussians to capture facilitatory and inhibitory contributions. Hence, we call this variant APC–2D–inh. Despite incorporating inhibitory shape contributions, Pasupathy and Connor (2001) reported “the average increase in *r* was moderate (0.07)” in APC–2D–inh compared with APC–2D. For this reason, we keep APC–2D as the representative tuning for comparison purposes. Finally, we compare SparseShape with the correlations of neurons in Alexnet (Krizhevsky et al., 2017).

An ideal comparison of our model results to the published results of the HMAX and APC models would require their original code, and neither model code is available. As a result, we extracted correlation coefficients of these models from their correlation histograms in Cadieu et al. (2007, their Fig. 5) and Pasupathy and Connor (2001, their Fig. 9), respectively. These authors did not publish the parameterizations for their model fit to each individual V4 neuron; and even if we had access to their code, there is no guarantee that we could find the parameter initialization that led to their published results. Comparison to their published results via extracted correlations remains the only option. Had we had access to their fit models, direct comparison between the models would be possible. For example, we could report MAE for HMAX and APC along with 2DSIL and SparseShape. Similarly, training on the same validation/train/test sets as those used to train/test HMAX neurons could provide a like-for-like comparison between SparseShape and HMAX. We had model shape neuron responses of 2DSIL that we used for comparison and computation of correlation coefficients for this model. Correlation coefficients of Alexnet were computed as follows: for each biological V4 neuron, we isolated a model cell across all convolutional layers of Alexnet with the largest correlation coefficient in response to the shape stimulus set. This procedure resulted in 109 model cells in Alexnet most similar in responses of the 109 Macaque V4 cells.

### Code accessibility

Our code is available on the Open Science Framework website: https://osf.io/u3zka/?view_only=f297fc30c24142d6829121e6bbab33de.

## Results

### Shape part selectivity

**Figure 4.**
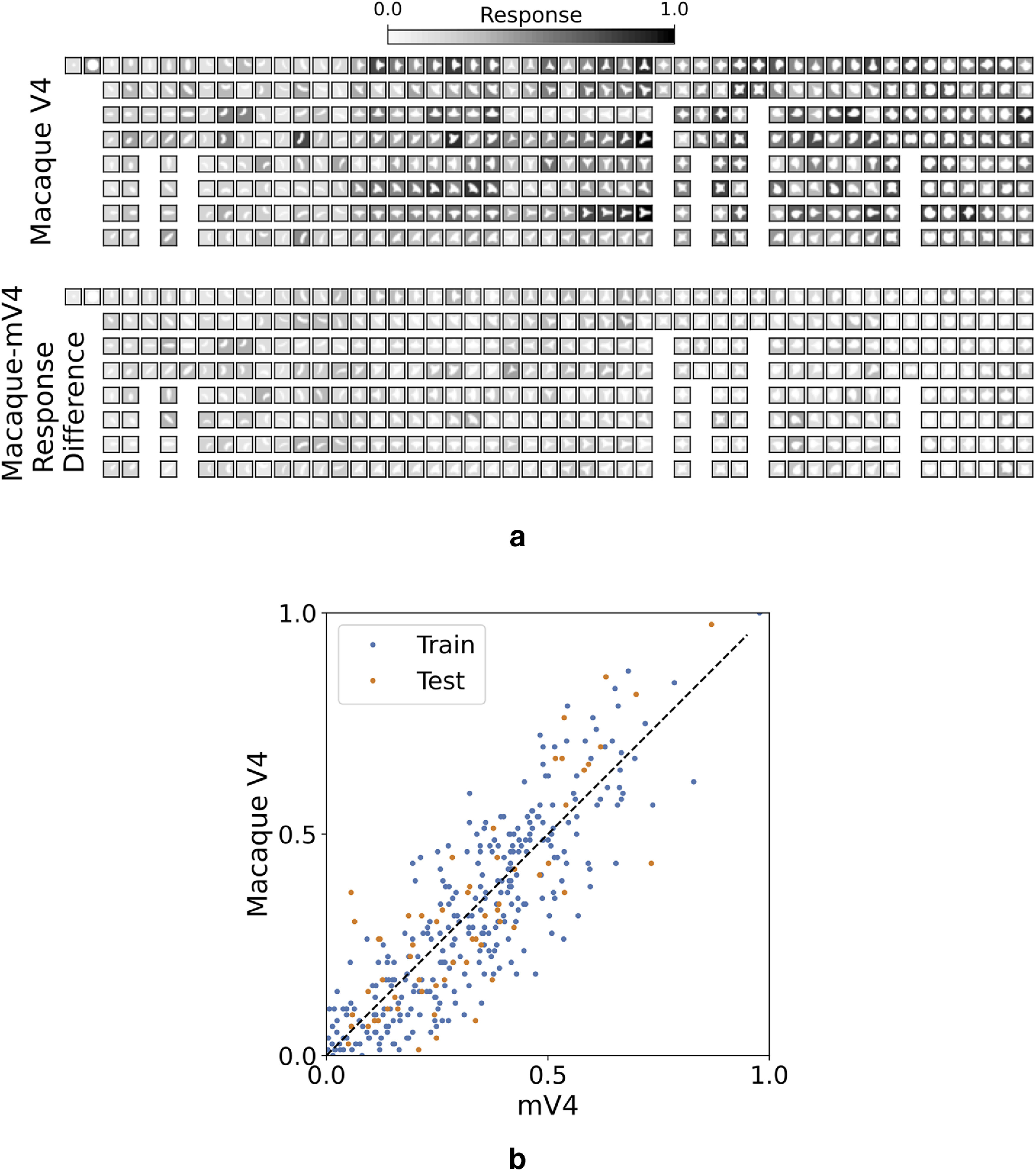
Responses of a learned mV4 cell. ***a***, Two rows Macaque V4 responses and response differences with mV4 to the stimulus set introduced by Pasupathy and Connor (2001). White icon in a square represents each shape. The intensity of the ground in each square indicates the response strength. Small response differences in the second row demonstrate a similar pattern of responses in the mV4 cell and the Macaque V4 cell. ***b***, Macaque V4 responses plotted against mV4 activations illustrate a strong correlation between these responses (*r* = 0.86 and *r* = 0.83 for train and test shapes, respectively).

**Figure 5.**
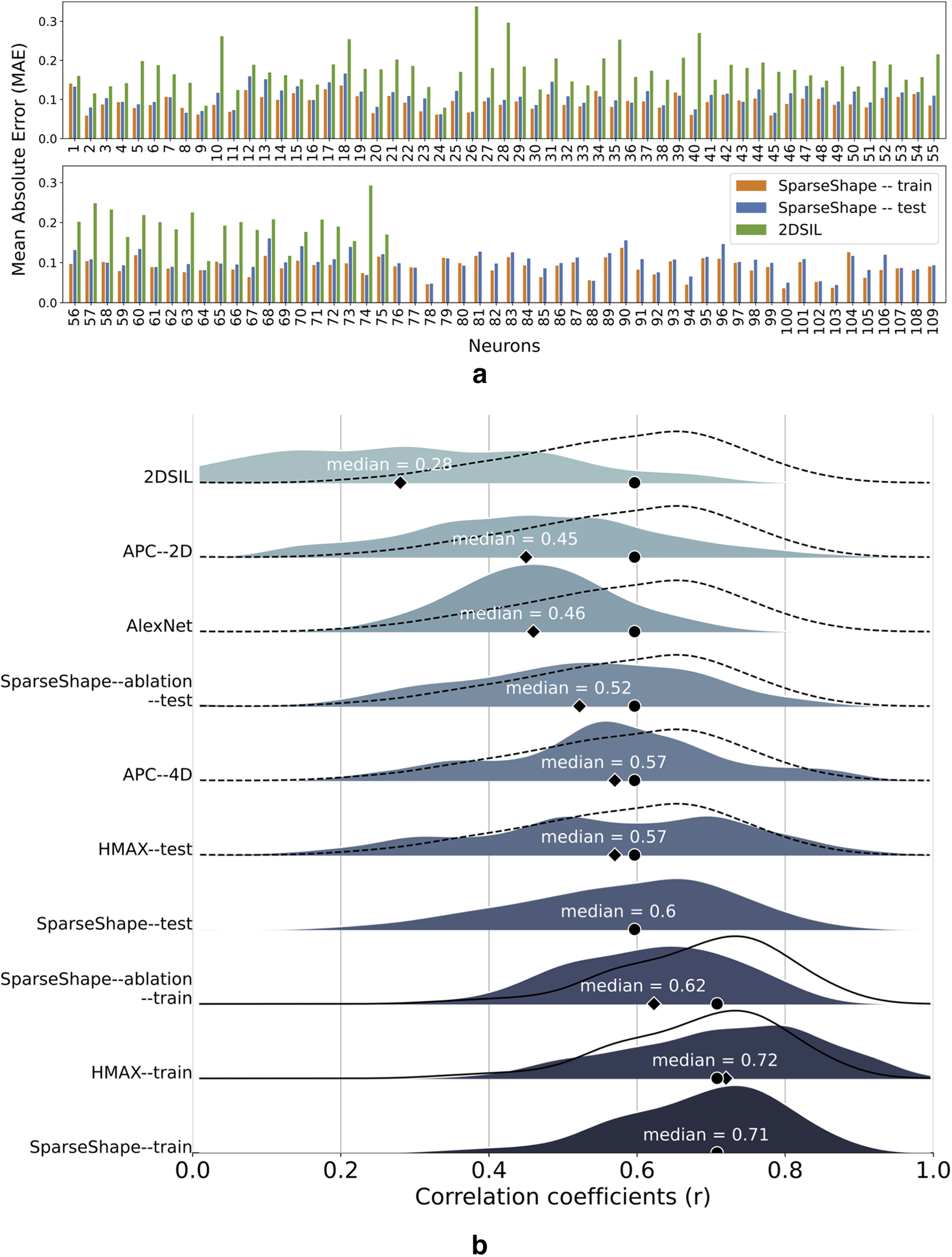
Model performance evaluation. ***a***, MAE in responses of model V4 cells for 2DSIL and SparseShape. Neuron errors are plotted in two rows for better visualization. SparseShape improves the performance measured as the mean difference of responses with biological V4 cells compared with 2DSIL. ***b***, Population distribution of correlation coefficients, *r*, for various V4 models plotted as a ridge-line plot in separate rows. Each model is indicated next to its distribution. The models are sorted according to their medians from bottom to top, with the exception of SparseShape–train. For easier comparison, SparseShape–train distribution is plotted as a solid line for HMAX–train and SparseShape–ablation–train rows and SparseShape–test distribution as a dotted curve in rows for all other models. The median for each distribution is denoted by a diamond shape, the value of which is written in white, and the median of Sparseshape–train/test distributions is identified with a black circle in each row. This plot demonstrates that SparseShape has comparable distributions to HMAX with better generalization capacity over the standard shape set and improves the correlation coefficients compared with other models. This plot also includes correlation distributions of an ablation study where we limited the contribution type in SparseShape to facilitatory ones to examine the effects of inhibitory shape parts to V4 responses. Comparing SparseShape and SparseShape–ablation distributions suggest inhibitory shape parts integration in V4. Among all the models, Alexnet and 2DSIL with smaller correlations compared with the rest of the models demonstrate a poor fit of their neurons to V4 data.

Figure 4*a* depicts responses of a Macaque V4 neuron to the standard shape set and response differences between Macaque V4 and its corresponding mV4 cell. Small response differences suggest similar selectivities in Macaque V4 and mV4 cells that are also confirmed with the strong correlations demonstrated in Figure 4*b*. To measure this similarity in the population, we computed the MAE separately for train and test shapes for all 109 Macaque V4 cells, illustrated in Figure 5*a*. This figure demonstrates a decrease of MAE in SparseShape versus 2DSIL in 75 Macaque V4 neurons. Additionally, the remaining 34 cells follow a similar trend in train and test MAE. The average MAE over all mV4 cells is 0.09 in SparseShape versus 0.18 in 2DSIL. MAEs for the rest of the models were not available; therefore, these models are missing in Figure 5*a*.

We computed the correlation coefficients, *r*, as a goodness-of-fit measure between Macaque and model cell responses following Pasupathy and Connor (2001) and Cadieu et al. (2007). While the APC model was fit based on responses to all the 366 stimuli, HMAX used a six-fold cross-validation and reported the average correlation coefficient. In other words, HMAX fits six models to each V4 cell and reports the average performance of the six models. Cross-validation correlations are not appropriate indicators of the generalization abilities of the model; and with six models learned for each neuron, it is unclear which must be considered as the true model of the cell. However, in absence of generalization data from HMAX, we compare SparseShape with their reported cross-validation correlations. SparseShape evaluation is based on separate train-test splits that were explained earlier. Figure 5*b* illustrates the correlation coefficient distributions for APC–2D, APC–4D (extracted from correlation histograms in Pasupathy and Connor, 2001, their Fig. 9), HMAX (extracted from correlation histograms in Cadieu et al., 2007, their Fig. 5), Alexnet (extracted from Pospisil et al., 2018, their Fig. 14), 2DSIL, and SparseShape. A ridgeline plot separates these distributions. For easier comparison, SparseShape–train/test distributions are plotted as solid and dotted curves, respectively, in rows of other models.

Despite the slight shift in the correlations distribution for HMAX–train compared with SparseShape–train depicted in Figure 5*b*, both models have comparable medians at 0.72 and 0.71, indicating a great overlap between the simulated populations. In contrast, SparseShape–test distribution is slightly shifted with a larger median at 0.6 compared with 0.57 for HMAX–test, demonstrating better generalization ability over the standard set for SparseShape. The impressive correlations and recovery of V4 selectivities that HMAX displays are based on the assumption that there are no intermediate computational stages between orientation (complex) neurons and 2D shape cells. This is in line with the way computer vision has viewed the problem of 2D shape over the past; it is not consistent with the available neurobiology, however, that shows a much richer connectivity (see Ungerleider et al., 2008) that would suggest this assumption is oversimplifying the network. This has a number of consequences. HMAX neurons are insensitive to the position of the shape within their RF as we demonstrated in Figure 1*c*, whereas V4 cells do exhibit sensitivity to shape position (Pasupathy and Connor (1999). An intermediate step of curvature sign between orientation selectivity in V1 and shape selectivity in V4 would have provided what is required to make these cells position aware, as our model shows. Second, the assumption leads to HMAX being a single-task network: it may provide a good fit to a particular set of V4 data, but when situated in a full network (e.g., detailed by Ungerleider et al., 2008, and many others), HMAX alone may not play the full role for which those neurons are responsible. Other perceptual functions, such as BO, figure-ground segmentation, localizing concave or convex shape elements, recognizing sharpness of shape, and more, would fall outside the range of HMAX.

Interestingly, Alexnet with a deep convolutional neural network architecture is outperformed by all other models, except for 2DSIL and APC–2D, demonstrating a poor fit of its units to V4 data.

The APC distributions are most interesting. Despite the fit to the whole standard set, both APC–4D and APC–2D distributions are shifted to smaller correlations compared with the SparseShape. Larger correlation coefficients in APC–4D versus APC–2D, as pointed out by Pasupathy and Connor (2001), suggest complex configuration encodings in V4 cells. Likewise, comparing APC–4D and SparseShape distributions suggests a complicated pattern of interactions between shape parts within V4 RFs, beyond a combination of three adjacent parts.

Pasupathy and Connor (2001) reported moderate improvement in correlation coefficients in their APC–2D–inh model by fitting two Gaussians with arbitrary contributions (facilitatory and inhibitory) in the APC space. To quote Pasupathy and Connor (2001), they suggested that “the two-Gaussian analysis is just one fairly simple approach, and it may be that another, more complex analysis would provide a much better description of shape tuning.” Despite this suggestion, the APC–2D model has been used in recent V4 studies (El-Shamayleh and Pasupathy, 2016; Popovkina et al., 2019), thus not capturing some of the details that APC–2D–inh might include. In contrast, the correlation coefficients of SparseShape versus those of both APC–2D and APC–2D–inh suggest that our model captures details that a simple two-Gaussian model cannot. For example, the supervised sparse coding step in SparseShape with no hard constraints imposed on the number of contributing shape parts provides a fairly simple approach to reveal a richer description of shape tuning in these neurons as explored next.

### Sparsity

**Figure 6.**
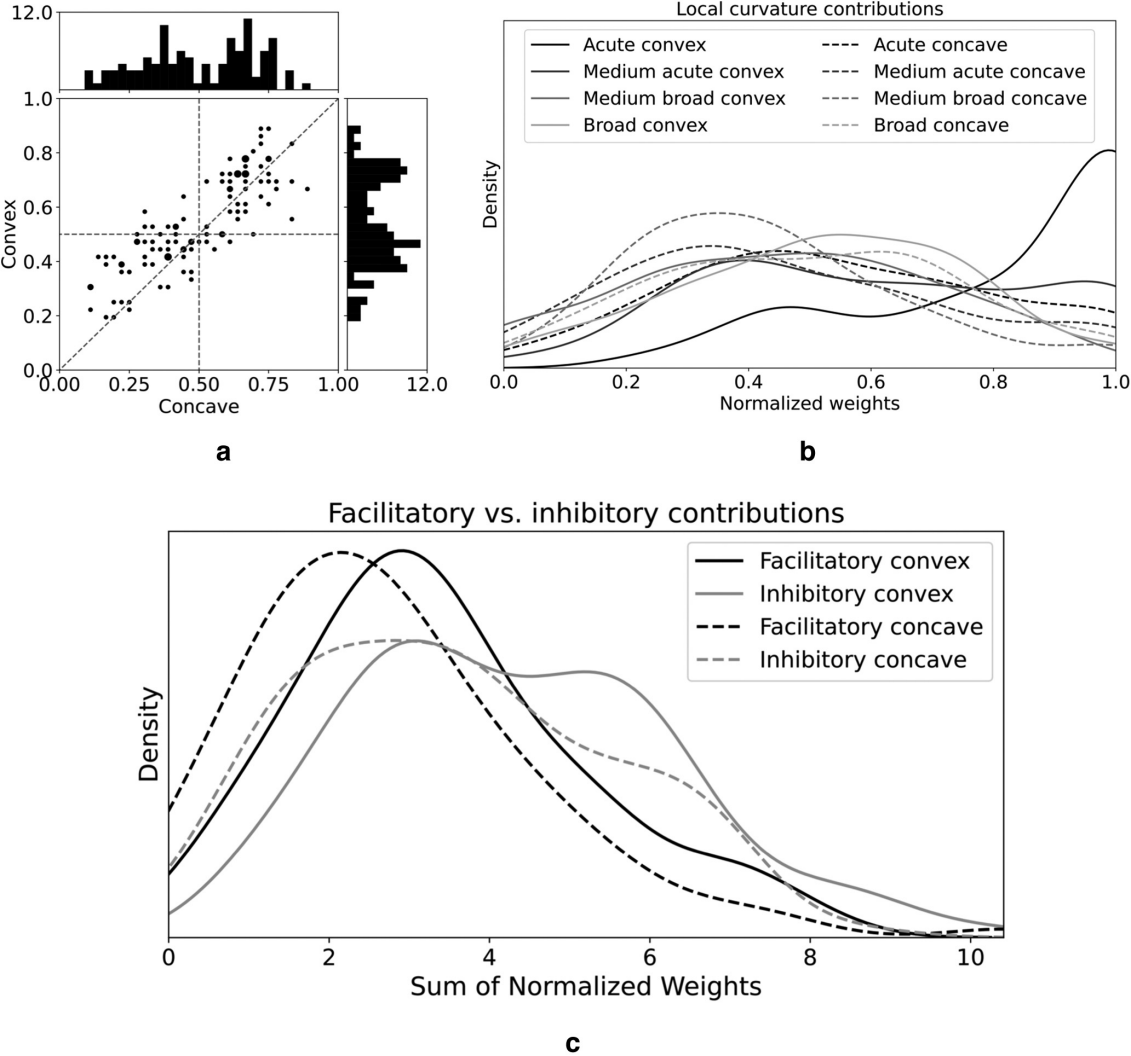
Sparsity and contribution type analysis. ***a***, For each neuron in the mV4 population, we calculated the percentage of nonzero convex and concave elements of the sparse code vector. Each dot represents a neuron. Dot sizes are proportional to the number of neurons matching the same sparsity. This figure demonstrates that more than a single or three curvature components, such as those modeled in APC–2D and APC–4D, contribute to V4 activations. Moreover, more dots above the identity line in this plot confirm previous observations of bias toward convexities in V4 neurons (Pasupathy and Connor, 1999; Carlson et al., 2011). ***b***, Distributions of normalized weights from mLocalCurv maps to mV4 neurons separated according to the eight modeled signed curvatures. Overall, all distributions overlap, except for the acute convex weights that peak at 1. This plot in conjunction with results from ***a*** represent a bias toward convexities in mV4 neurons. ***c***, Facilitatory versus inhibitory weight distributions. The distribution of facilitatory and inhibitory contributions from shape parts greatly overlaps, with a slight shift for facilitatory convex weights, suggesting that both types of contributions determine V4 responses.

To measure the number of parts contributing to mV4 responses in SparseShape, we examined the sparsity of the learned sparse code vector as the percentage of its nonzero elements separately for convex and concave parts for each mV4 cell. Figure 6*a* demonstrates the sparsity distribution for the population with each neuron displayed as a dot. This distribution suggests contributions from more than a single or even three shape parts to mV4 responses. Additionally, convex sparsity median at 0.53 compared with 0.5 for concave sparsity implies a bias toward convexities in agreement with the observations reported previously (Pasupathy and Connor, 1999; Carlson et al., 2011).

With more convex parts contributing to mV4 responses, one possibility is smaller convex weights compared with fewer but larger concave weights to compensate for this imbalance. To evaluate this possibility, for each neuron, we normalized the sparse code vector with maximum set at 1. Then, we took the largest weight from each signed curvature type (each mLocalCurv map), resulting in an eight-element vector for each neuron. Considering the population distribution over each element of this vector, a shift to larger weights is expected from signed curvature components with substantial contributions to mV4 responses. Interestingly, as illustrated in Figure 6*b*, all distributions are relatively overlapping, except for that of acute convexities peaking at 1, confirming a bias toward convexities in the learned model.

### Contribution types

If V4 neurons indeed integrate V2 responses in both excitatory and inhibitory manners, models limited to facilitatory weights neglect accounting for important contributions to V4 responses. In SparseShape, both types of contributions are explored during learning and accounted for in the model. To assess the effect of contribution type to mV4 responses, we considered the population distribution of the sum of facilitatory and inhibitory weights in the normalized sparse code vector (max at 1). Interestingly, as depicted in Figure 6*c*, the distributions plotted separately for convex and concave parts demonstrate larger inhibitory effects from both convex and concave parts, suggesting that excluding inhibitory contributions result in an incomplete understanding of V4 shape processing. To further investigate the effect of inhibitory contributions, we performed an ablation study in which we restricted part contributions to excitatory ones in SparseShape. With this imposed constraint, as demonstrated in Figure 5*b*, SparseShape–ablation–train and test correlation distribution medians are decreased to 0.62 and 0.52, respectively. These results, compared with train/test medians at 0.71 and 0.6 in SparseShape with inhibitory part contributions, highlight the importance of inhibition in V4 responses.

### RF visualization

**Figure 7.**
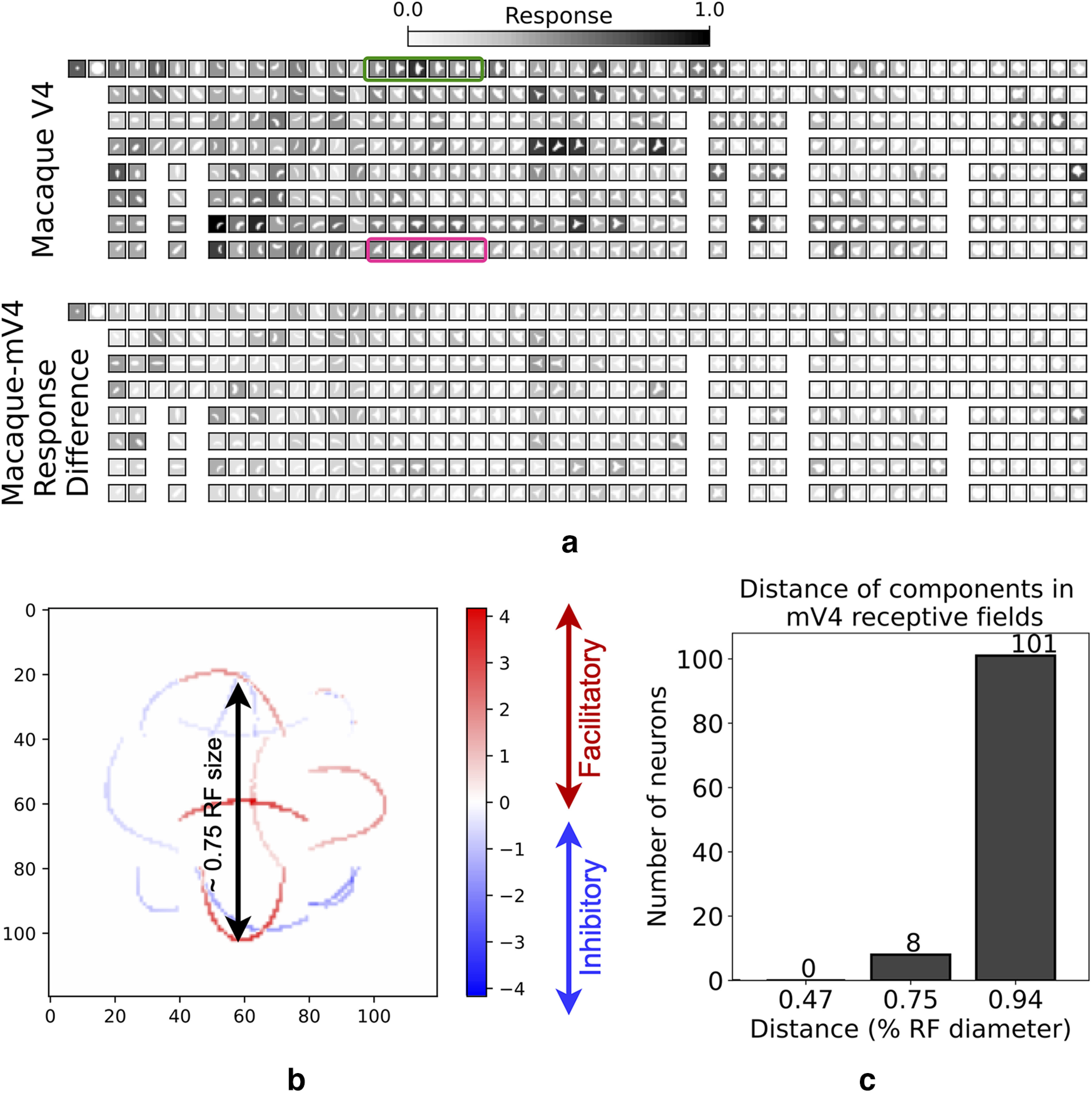
Recovered mV4 RFs. ***a***, Responses of an mV4 cell are shown. The shapes inside the green box evoke strong activations in the biological neuron. Same shapes but rotated at –45 degrees inside the magenta box evoke much weaker activations in this cell. ***b***, The recovered RF of the same neuron whose responses are shown in ***a***. Red and blue represent facilitatory and inhibitory contributions, respectively. Shapes with medium/broad convexities at the top, bottom, and right part of the RF evoke strong activations in the cell. In contrast, medium/broad convexities in the bottom right and left parts of the RF inhibit neuronal responses. The recovered RF displays that shape parts from the spatial extent of the neuron's RF, as far as 0.75 × RF size, are combined to account for its selectivity. Such long-range interactions between shape parts were not captured in the APC model because of fitting a single Gaussian to neuron responses. ***c***, Population histogram of shape part distances within mV4 RFs. The majority of mV4 cells integrate shape parts that are apart at least as far as three-fourths of the RF diameter, suggesting that V4 neurons incorporate V2 responses from the full span of their RF. The 0.95 × RF diameter is for shape parts on the diagonal of the simulated square-shaped RFs.

Visualizing the recovered RFs reveals shape selectivities in each mV4 cell. In SparseShape, modeling a hierarchy of representations that include intermediate- and higher-level feature of signed curvature makes explaining the recovered RFs effortless. For instance, Figure 7 shows an example neuron response along with a visualization of its recovered RF. Figure 7*b* demonstrates selectivity to mild and broad convexities in the top/bottom/right part of the RF. Responses of this cell are inhibited with appearance of mild convexities on bottom right and bottom left parts of the RF, respectively. The recovered RF can be qualitatively verified with the Macaque V4 responses in Figure 7*a*. For example, the shapes within the green rectangle in Figure 7*a* have three convex parts on facilitatory positions causing relatively strong activations of the cell. The same shapes rotated with two convex parts within the inhibitory parts of the RF (encompassed with a magenta rectangle) invoke weaker activations in the neuron.

Figure 7*b* presents an interesting observation: the contributions to this neuron's responses are from the full spatial extent of the cell with interacting shape parts separated at almost 0.75× RF size. To quantify the extent of shape part interactions within the RF for each neuron, we computed the distance of all pairs of contributing parts in the RF and took the maximum distance among all parts. Figure 7*c* shows the mV4 population distance histogram indicating that the majority of neurons integrate shape parts from at least as far as three-fourths of their RF diameter. This observation suggests that a single-Gaussian prior is far too limiting to capture all the factors contributing to V4 responses.

Visualizing the pattern of learned weights provides a better picture of selectivities in V4 cells. It can also be used as a tool for forming testable hypotheses for shape selectivity in these neurons. For example, if further mining of the learned weights in these neurons reveals RF areas dedicated to facilitatory/inhibitory shape parts, the analysis could assist in introducing models similar to the difference of Gaussians or Gabor models explaining orientation selectivity in simple cells in V1. Such an attempt, if made, will be a step toward better understanding of 2D shape processing in V4. We left further analysis of the learned weights for a future work.

### Invariance

**Figure 8.**
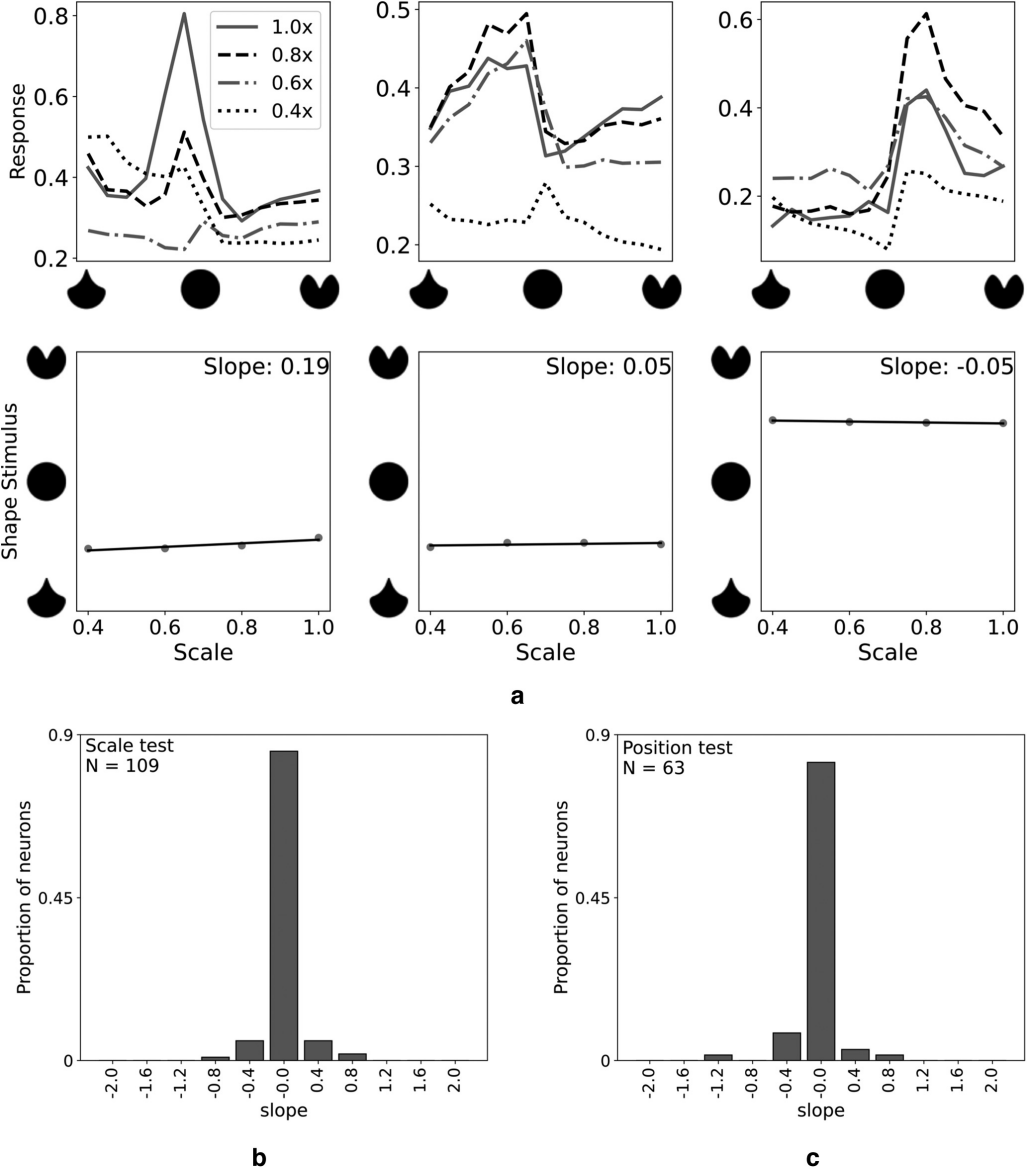
The learned mV4 neurons exhibit scale and position invariance. ***a***, Scale invariance in responses of mV4 neurons. Top row represents responses of three example mV4 cells to stimuli of various scales with their corresponding tuning centroid slope as a function of scale shown in the bottom row in this panel. These plots suggest that mV4 cells exhibit “normalized” curvature encoding similar to Macaque V4 neurons. The same legend applies to all plots on the first row. ***b***, Tuning centroid slope distribution for the population of mV4 neurons in SparseShape for the scale test. ***c***, Slope distribution for the position test. ***b***, ***c***, The learned mV4 neurons show similar scale and position invariance properties as Macaque V4 neurons (El-Shamayleh and Pasupathy, 2016) despite training on a single scale.

El-Shamayleh and Pasupathy (2016) reported a normalized curvature encoding in V4 cells. We probed our mV4 cells to determine whether the learned selectivities encode normalized or absolute curvature (for definitions and details of their experimental setting, see El-Shamayleh and Pasupathy, 2016). For this purpose, we prepared shapes of varying scales similar to those used by El-Shamayleh and Pasupathy (2016) and evaluated systematic shifts in tuning centroids in mV4 responses. Briefly, if these neurons exhibit scale invariance, their tuning peak would not shift with changes in scale. Therefore, measuring the shift in tuning centroids as a function of scale would result in slopes close to zero. To examine whether the observed invariance in responses could be attributed to the shift in the position of boundary conformation, we tested our mV4 responses to changes in stimuli position within RF, similar to El-Shamayleh and Pasupathy (2016). Figure 8*a* demonstrates a few examples of mV4 neuron responses with scale-invariant selectivities as indicated by the small slopes. Population histograms of tuning centroid slopes, depicted in Figure 8*b*, *c*, suggest that the learned mV4 neurons exhibit invariance properties similar to Macaque V4 cells (compare with histograms in El-Shamayleh and Pasupathy, 2016, their Figs. 5, 9).

Results from this experiment are interesting in that our mV4 neurons were trained with a single scale stimulus set. However, the learned weights in SparseShape generalized to account for these variations in responses of Macaque V4 cells.

## Discussion

Our goal was to understand the shape signal transformation in the ventral stream up to V4. The present work used findings of the various visual areas involved in shape processing in this stream along with geometry and machine learning to propose a step-by-step explanation for this transformation. We refrained from using end-to-end deep neural networks because explaining what is learned in each layer of these deep architectures is still an open problem. Instead, we took an alternative approach and proposed a mechanistic model with explicit algorithmic steps that could explain the role each component plays in the shape signal transformation from orientation selectivity to abstract representations of signed curvature and part-based selectivity in V4. We stood on the shoulder of experimental findings of the brain, where possible, and designed the model and set its parameters accordingly. Only in absence of such knowledge, we gained from a machine learning algorithm to unlock new information hidden in existing V4 data: our results indicate contributions from multiple parts from the full spatial extent of RF in both facilitatory and inhibitory manners.

Throughout this manuscript, we made the effort to demonstrate the important role both curvature magnitude and sign representations play in achieving a signed curvature encoding. Recently, Pasupathy et al. (2020) referred to V4 representations as “object-centered.” We, instead, used the geometric term “signed curvature” to emphasize selectivity to both curvature magnitude and curvature sign in V4 neurons as was initially reported in Pasupathy and Connor (1999, 2001, 2002). Object-centered V4 selectivities refer to awareness to inside-outside (BO) and omit the curvature magnitude selectivity in these neurons.

Similarly, when it comes to a model of V4, those previous models that omit incorporating both curvature components cannot achieve a signed curvature encoding and, hence, are lacking with respect to our current understanding of shape processing in V4. Even with their impressive population distribution of correlation coefficients, HMAX falls under this category. Without incorporating curvature sign, it is not surprising that HMAX failed to exhibit similar levels of invariance to changes in position as V4 neurons; with an example, we showed (Fig. 1*c*) that the same configuration of oriented edges recovered in HMAX could be a convex or concave segment on the bounding contour of a shape depending on the shape position in the RF. Additionally, the HMAX hierarchy skips intermediate processing stages and jumps from orientation-selective neurons to shape selectivity in V4. As a result, some of the recovered RFs look like an ensemble of oriented edges that are difficult to interpret. Such an approach fails to explain how the shape signal transforms in the ventral stream. In contrast, with explicit modeling of intermediate representations, not only did we achieve a signed curvature encoding, but also provided a step-by-step explanation for the development of this representation in the ventral stream.

Compared with other models, SparseShape outperformed 2DSIL, Alexnet, and APC. Although APC was fit to all the shapes in the standard set and we trained our model with only 60% of shapes, SparseShape–train outperformed APC–4D. This mismatch in performance suggests that more than three juxtaposed shape parts contribute to V4 responses. This observation was confirmed in our sparsity experiment and was evident in the visualized RFs: V4 neurons integrate shape parts from the full spatial extent of their RF, which is more efficient than limiting selectivity to a small portion of the RF.

Not only long-range interactions of shape parts within the RF emerged from relaxing the priors compared with APC, a combination of facilitatory and inhibitory contributions also appeared in the recovered RFs. All previous models of V4 with direct comparison with recordings from the study by Pasupathy and Connor (2001) were limited to facilitatory contributions of parts. However, the relaxed priors in SparseShape revealed more complicated part-based selectivities in V4 neurons. Facilitatory and inhibitory part contributions in SparseShape reiterate the findings of Brincat and Connor (2004) in IT; therefore, it is not surprising to find that V4 neurons follow suit.

In SparseShape, the signed curvature encoding relies on both endstopping and BO. Discoveries of both types of representations in the ventral stream lend additional support to the development of a signed curvature encoding in this stream. Responses of V4 cells to shapes that are extended beyond the RF (Pasupathy and Connor, 1999) and similar responses in BO cells suggest that similar mechanisms govern activations in both neuron types. We acknowledge that findings of Bushnell et al. (2011) suggest against BO contributing to V4 responses. Their argument is on the basis of time course in BO and V4 neurons. SparseShape incorporates the RBO network to generate the BO signal. The RBO hierarchy is designed based on early recurrence from MT to BO neurons and provides an explanation for the early divergence of responses in BO cells (Mehrani and Tsotsos (2021). Whether this early divergence is relayed to V4 cells, earlier than reaching half-max responses in BO as used for the argument in Bushnell et al. (2011), or a direct recurrence from MT provides side-of-figure information to V4 neurons remains to be further investigated. The message here, however, is that both curvature magnitude and curvature sign (perhaps from similar mechanisms that give rise to BO) are essential to forming a signed curvature encoding. How and where from in the brain this information is provided to V4 is another question that is put forth to be investigated in the future.

Our proposed model revealed hidden information, more than those previously reported, in existing V4 data. These findings impart new insights into shape processing in V4 that require further testing and investigation in biological V4 neurons. For example, examining biological V4 responses by removing shape parts allows testing for facilitatory versus inhibitory contributions, multipart selectivity, and the extent of part integration within the RF. Removing a shape part inevitably disturbs the curvature sign in a geometric sense and might make the experimental design a challenge in this case. Luckily, Zhang and von der Heydt (2010) found that the side-of-figure preferences are maintained in BO neurons even when boundary fragments are removed from Cornsweet shapes. Additionally, the RBO network could explain illusory contours. Together, these findings provide a unique tool to further test the signed curvature encoding in the ventral stream and to enhance our understanding of contributions of shape parts to V4 responses. Specifically, if V4 cells receive inside-outside information that originates from BO cells, converting the standard stimulus set to Cornsweet shapes allows removal of shape parts without disturbing the overall inside-outside and consequently the signed curvature signal for the shape. Then, the findings from the present work can be examined in biological V4 cells. For example, removing a part that inhibits a cell's responses according to the model is expected to increase the neuron's activations when all other shape parts are intact. Such a study is different from those in which V4 responses to occlusion were examined (Bushnell et al., 2011; Fyall et al., 2017). In the occlusion studies, the shape is occluded with another form which disturbs BO and consequently signed curvature representations. In contrast, with the suggested experimental design, there is no occlusion but absence of a part, making testing the effect of each individual part a possibility without changing the overall shape representation.

Our proposed model can be further extended. For example, in the present model, we combined responses of border- and edge-selective mBO cells. Maintaining those signals allows modeling a wider variety of mLocalCurv neurons and consequently modeling V4 responses to outline/filled shapes (Popovkina et al., 2019). Similarly, adding neurons selective to texture could lend insight into joint shape and texture processing in this area (Kim et al., 2019). Also, we did not implement mechanisms to represent inflection points (zero curvature), such as straight lines at mV4 level. Adding connections from mV1 layer to mV4 can handle such cases. Such extensions are left for future work. Finally, interesting recent findings of shape processing in V4 revealed both flat and solid shape selectivity in V4 (Srinath et al., 2021). Here, we focused on modeling neuron responses in V4 modules dedicated to 2D shape processing. However, the recent V4 findings open exciting new possibilities for future extensions of SparseShape.
